# Taking it with a grain of salt: tolerance to increasing salinization in *Culex pipiens* (Diptera: Culicidae) across a low-lying delta

**DOI:** 10.1186/s13071-024-06268-8

**Published:** 2024-06-10

**Authors:** Sam Philip Boerlijst, Antje van der Gaast, Lisa Maria Wilhelmina Adema, Roderick Wiebe Bouman, Eline Boelee, Peter Michiel van Bodegom, Maarten Schrama

**Affiliations:** 1 Center for Environmental Research Leiden, Department of Environmental Biology, Leiden University, Einsteinweg 2, 2333 CC Leiden, The Netherlands; 2 Division of Inland Water Systems, Deltares, 177, 2600 MH Delft, The Netherlands; 3 Hortus Botanicus Leiden, 9500, 2300 RA Leiden, The Netherlands; 4 Naturalis Biodiversity Center, 9517, 2300 RA Leiden, Netherlands; 5 Institute of Biology Leiden, Leiden University, 9505, 2300 RA Leiden, Netherlands

**Keywords:** Adaptation, *Culex pipiens*, Environmental change, Mosquito, Population dynamics, Oviposition experiments, Salinization

## Abstract

**Background:**

Salinity, exacerbated by rising sea levels, is a critical environmental cue affecting freshwater ecosystems. Predicting ecosystem structure in response to such changes and their implications for the geographical distribution of arthropod disease vectors requires further insights into the plasticity and adaptability of lower trophic level species in freshwater systems. Our study investigated whether populations of the mosquito *Culex pipiens*, typically considered sensitive to salt, have adapted due to gradual exposure.

**Methods:**

Mesocosm experiments were conducted to evaluate responses in life history traits to increasing levels of salinity in three populations along a gradient perpendicular to the North Sea coast. Salt concentrations up to the brackish–marine transition zone (8 g/l chloride) were used, upon which no survival was expected. To determine how this process affects oviposition, a colonization experiment was performed by exposing the coastal population to the same concentrations.

**Results:**

While concentrations up to the currently described median lethal dose (LD_50_) (4 g/l) were surprisingly favored during egg laying, even the treatment with the highest salt concentration was incidentally colonized. Differences in development rates among populations were observed, but the influence of salinity was evident only at 4 g/l and higher, resulting in only a 1-day delay. Mortality rates were lower than expected, reaching only 20% for coastal and inland populations and 41% for the intermediate population at the highest salinity. Sex ratios remained unaffected across the tested range.

**Conclusions:**

The high tolerance to salinity for all key life history parameters across populations suggests that *Cx. pipiens* is unlikely to shift its distribution in the foreseeable future, with potential implications for the disease risk of associated pathogens.

**Graphical Abstract:**

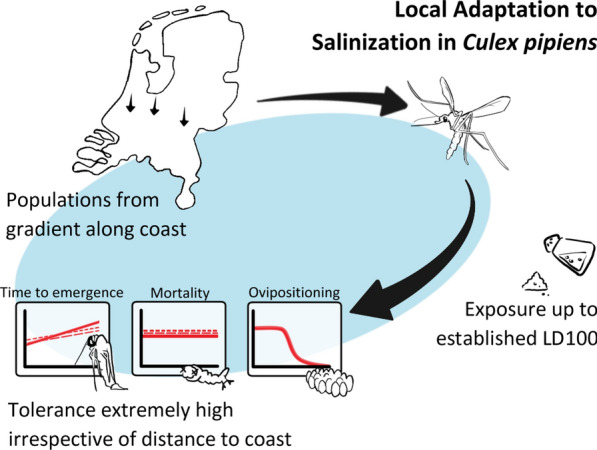

**Supplementary Information:**

The online version contains supplementary material available at 10.1186/s13071-024-06268-8.

## Background

Salinization of fresh water in coastal areas, especially in low-lying deltas, is a natural process that is currently exacerbated by anthropogenic drivers, such as climate change-induced sea level rise, land subsidence, and saline groundwater seepage, intensified by the removal of overlying fresh water [1]. Saltwater infiltration is commonly acknowledged to negatively affect agricultural yield and freshwater ecosystem services [2]. The underlying physical processes of salinization are relatively well described [3, 4], and animal diversity at large is understood to decrease under transitory conditions [5]. However, little is known about the direct and indirect effects of salinization on animal populations inhabiting (currently freshwater) ecosystems in deltas, especially for species that are disease vectors.

The cosmopolitan house mosquito *Culex pipiens* species complex is a known vector for a variety of pathogens, including West Nile virus, Usutu, and avian malaria [6–9]. It has a wide habitat tolerance, ranging from clean rainwater-filled containers to strongly polluted temporal water bodies, such as ground puddles, and even manure tanks [10, 11]. Similar to other mosquito larvae typically associated with fresh water, it accumulates organic osmolytes to combat ionic pressure instead of active ion transport [12] and is known to be quite vulnerable to changes in salinization relative to other mosquito species [13–15], with a median lethal dose (LD_50_) of 4 g/l and a lethal dose (LD_100_) of 6–10 g/l chloride for acute salinity stress [15–17].

Although a variety of responses to salinization exist among invertebrates [12], general trends exist in the whole invertebrate community. Salinization has been shown to shape insect community structures, negatively affecting diversity [18, 19] via decreased food availability [20, 21]. Although mosquitoes have previously been described to react quite similarly [5, 22], it has also been hypothesized that their short generation time (when compared to that of many other macrofauna species, including their predators [23]) might enable mosquitoes to adapt more rapidly [24–26]. This could subsequently cause a relative increase in population size in transitory systems due to the alleviation of predation pressure and the relative increase in food resources [19]. Such a fast adaptation rate is observed for a variety of other stressors, such as pesticides [27–29]. These adaptations are similar to the response to salinization, i.e., by affecting the excretion of harmful compounds [12, 30]. This renders it likely that mosquitoes are better able to adapt to increasing salinity than other insect species.

Salinization affects mosquito habitat quality and may thus reduce larval survival. However, this depends on how well the larvae are adapted to temporary (i.e., flooding) and continuous salinization events and processes, causing species-specific effects [15]. These adaptations in osmoregulation include physiological (reduced surface area of anal papillae or active transport of ions) [31, 31, 32] and behavioral adaptations (increased metabolism and uptake of organic compounds in hemolymph) [32–37], resulting in tolerance that changes across life stages [38] and differs between sexes [39]. Namely, female mosquitoes tend to be less strongly selected for early maturation, which may lead to prolonged exposure to stress as compared to males [40]. With time, adaptation to salinization has caused species-specific preferences during oviposition [40–43], further shaping mosquito community composition.

At the population level, commonly considered intolerant species such as *Cx. pipiens* sensu lato (s.l.) might be affected by salinization in a variety of ways. Salinization might cause (i) no change when tolerance via for instance plastic behavior proves sufficient, (ii) local extinction of the species if tolerance is insufficient, (iii) displacement when unfavorable conditions are perceived during ovipositing, or (iv) local adaptation leading to possibly increased tolerance due to gradual, continuous exposure.

This study aimed to evaluate whether (local) adaptation to salinization occurred, by quantifying and comparing the tolerance of *Cx. pipiens* populations along a gradient from coast to inland. We expected increasing levels of adaptation (i.e., lower mortality, more rapid development, and a balanced sex ratio) closer to the coast as a result of gradual exposure. To this end, we performed a mesocosm experiment. We varied concentrations from 0 to 8 g of chloride per liter with intervals of 2 g, i.e., from fresh water to the predicted maximum inland surface water concentration of 7.5 g/l Cl^−^ [44], or the brackish-marine transition zone [45], at almost half the concentration of seawater.

## Methods

### Collection and rearing of experimental populations

*Culex pipiens* egg rafts were collected during the 2 days prior to the start of an experimental round from one set of naturally colonized black plastic mesocosms in peri-urban areas of the cities of Leiden, Utrecht, and Nijmegen, representing coastal (7 km to sea), intermediate (43 km to sea), and inland (108 km to sea) mosquito populations, respectively. All populations were collected at similar altitudes (2–5 m above sea level [asl]). For this purpose, the mesocosms were filled with 6 l of hypertrophic water (100 mg N-total), after which they were placed under tree cover. The larvae were subsequently allowed to hatch in 50 ml Falcon tubes, where they were kept at ambient temperature until the start of the experiment. Previous pilot studies have indicated that this type of experiment attracts *Cx. pipiens* and *Culiseta annulata* only [40, 46]. The collected egg rafts were distinguished from those of *Cs. annulata* by their difference in size [47, 48].

### Experimental setup

The setup consisted of 45 white plastic 12 l mesocosms, each with a 200 W aquarium heater. The experiments were conducted under standardized outdoor conditions [40] at the Hortus botanicus, Leiden, the Netherlands. The aquarium heaters were programmed at a minimum temperature of 20 °C for optimal development, while allowing for natural fluctuations, so that the development was representative of field conditions during the peak of the Dutch mosquito season [40, 49, 50]. Namely, as increased temperature heightens metabolism, ion uptake and transport may be increased, making it imperative to work under such conditions.

All 45 mesocosms were filled with 8 l of dechlorinated tap water (maintained at a constant level during the experiments), a natural concentration of microbes, a high concentration of nutrients, and a specific concentration of sea salt (Jozo, Rotterdam, the Netherlands). For the natural concentration of microbes, 1 l of water from a local lake was filtered per liter of tap water using a 250 μm plankton net and 53 μm collector. The high concentration of nitrogen prevents food from being a limiting factor and thus minimizes cannibalism (Koenraadt and Takken, 2003). This was achieved by adding 20 mg/l N in the form of dry cow manure (2.4% N, 1.5% P_2_O_5_, and 3.1% K_2_O) to the water. The mesocosms were randomly allocated to five increasing concentrations of commercially available sea salt—0 g/l, 2 g/l, 4 g/l, 6 g/l, and 8 g/l Cl—and split into two rounds of experiments due to spatial constraints, which are described below. The treatments were representative of fresh water [51], the highest measured salinity in a Dutch ditch [52], the LD_50_ [15], the highest measured salinity in seepage water [52], and the highest reported LD_100_ for *Cx. pipiens* [15], respectively (Table 1). In the first round, 0 g/l, 2 g/l, and 6 g/l Cl^−^ were used, and in the second round, 0 g/l, 4 g/l, and 8 g/l Cl^−^ were used.

**Table 1:** Conversion table of salinity treatments for chloride and total salt concentrations

	Chloride			Total salts		
	g/l (‰)	ppm	%	g/l (‰)	ppm	%
Fresh water	0.0	0	0.0	0.0	0	0.0
Maximum ditch	2.0	2002	0.2	3.6	3604	0.4
LD_50_	4.0	4005	0.4	7.3	7308	0.7
Maximum seepage	6.0	6007	0.6	11.0	11,013	1.1
LD_100_	8.0	8009	0.8	14.6	14,617	1.5
Typical seawater	18.9	18,921	1.9	34.5	34,539	3.5

*ppm* parts per million

For each concentration, a mixture of water, microbes, nutrients, and sea salt was prepared [40, 46], and salt was added over the course of 4 days in equal parts to limit osmotic stress to the microbial community. The mixture was thereafter covered with fine mesh (0.1 mm) to prevent additional colonization and was subsequently left to acclimatize for a period of 2 weeks. After the acclimation period, the water was divided over the experimental mesocosms using a 500 μm sieve to filter out any detritus and macroinvertebrates. After filtering, 100 second-instar larvae were added, and the aquarium heaters were turned on. Allocation of the populations and saline concentrations was performed in a Latin square, leading to five replicates for each population–concentration combination. During the experiment, the mesocosms were once again closed off using mesh to prevent predation and colonization from the outside and to ensure that the emerged mosquitoes could not escape. Temperature, chlorophyll *a* concentration, turbidity, and conductivity were measured as potential covariates using a Hach HQ40d multi-parameter meter and Turner Designs AquaFluor. Before the second round of the experiment, the original mixtures were collected, and the concentrations were increased from 2 g/l to 4 g/l and from 6 g/l to 8 g/l. The mixtures were once again left to acclimatize and were subsequently allocated to a new Latin square.

### Measurements of population parameters

Larval development was measured 5 days a week. First, the water was stirred clockwise once with a 400 mm-wide Φ 200 μm sieve to create a circular water flow and prevent the larvae from diving. The sieve was subsequently used to collect the larvae by fully submerging the sieve and moving it counterclockwise twice. All collected larvae were morphologically characterized to developmental stage using the size of the head capsule as a morphological indicator [53]. The identifications were compared daily with a previously reared reference collection of *Cx. pipiens* developmental stages. The procedure was repeated up to five times until at least 20 larvae were sampled.

Pupae were collected daily, after which they were allowed to emerge in 50 ml Falcon tubes. Sex was determined based on characteristics including plumose/pilose antennae and the length of the palps [53]. The proportion of total survival was determined by dividing the number of emerged adults by the original density of 100 larvae. The proportion of survival, used for visualization, was calculated by subtracting the mean of the control per population from the absolute survival rate. The time to pupation was determined after completion of the experiment. Time to pupation was defined as the interval between the start of the experiment and the first day upon which at least 50% of the subsampled larvae had turned/developed into pupae. The median time to emergence was determined by calculating the interval between the start of the experiment and the capture of 50% of the emerged adults. When no more pupae or adult mosquitoes were found for two subsequent days in a mesocosm, it was assumed that no living mosquitoes remained, and the mesocosm was closed off.

### Ovipositioning behavior

The ovipositioning behavior of the coastal population was determined in a separate experiment at the Hortus botanicus Leiden, the Netherlands. Five clusters—each consisting of one black, plastic 8 l bucket for each of the five salt concentrations—were placed around the botanical gardens at a distance of at least 58 m from each other to prevent the clusters from interfering with each other. The water, microbial community, and salinity levels were prepared as described in the previous section. Ovipositioning behavior was recorded by daily counts of egg rafts per mesocosm for a total of 12 days. Encountered egg rafts were removed to minimize the positive feedback caused by their presence [54].

### Statistical analyses

All data were analyzed in R version 4.2.2 [55]. Variance across experimental rounds was normalized based on the observed variance across the experimental rounds per population per salinity. Log–logistic regression was used to determine the LD_50_ and LD_100_ using the drc package [56]. Linear mixed-effects models were used to test for (normalized) differences in survival, development time (to pupation and emergence), and sex ratio across the different salinity levels. The salinity level, population, experimental round, average turbidity, conductivity, and chlorophyll *a* concentration were included as covariates. The individual mesocosms were included as random effect. The effect on ovipositioning behavior was similarly explored; a linear mixed model was applied using salinity level as main effects and day and location as random variables. All models (Additional file 1: Table S1) were optimized by the Akaike information criterion using stepwise regression with backward elimination. Dependent variables were tested for normality and assessed using quantile–quantile plots and Levene's test (*P* = 0.05).

## Results

### Effect of salinity on total proportion of survival

**Fig. 1:**
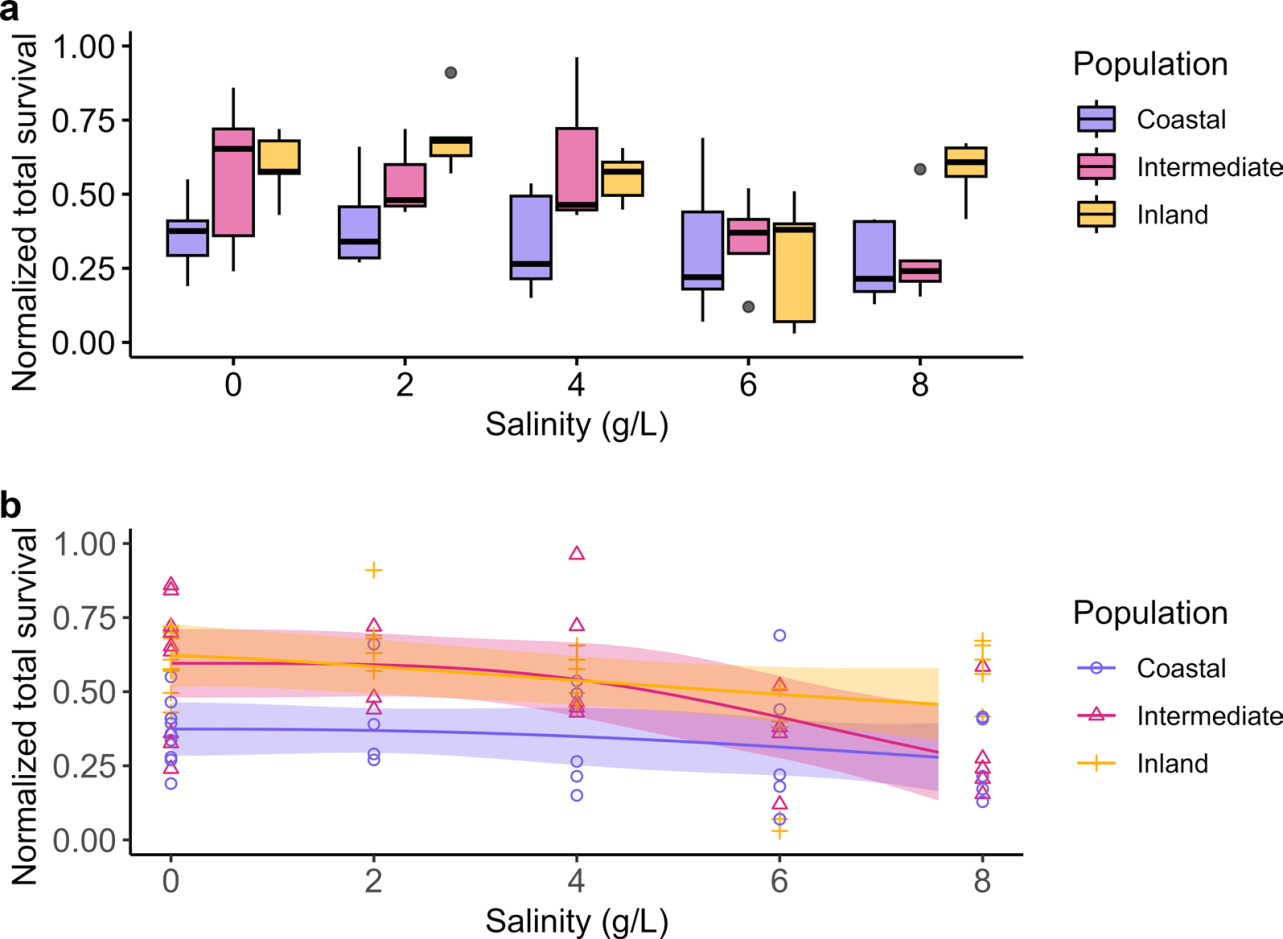
Proportion of normalized total survival per population across increasing salinization levels as **a** boxplot with outliers as dots and **b** dose–response curve with standard error. Total survival is depicted as the number of emerged adults at the end of the experiment as a fraction of the initial number of larvae

The total proportion of survival decreased with increasing salinity for all populations (*F*_(4,85)_ = 5.60, *P* < 0.001, partial η^2^ = 0.281), with 18%, 42%, and 20% (*p* < 0.001, *P* = 0.005, and *p* = 0.001 for coastal, intermediate, and inland, respectively; Fig. 1) from 4 g/l onward (Additional file 1: Tables S1, S2). Differences in slope were detected between the coastal and intermediate populations (*t*_(30,27)_ = −2.51, *P* adj < 0.001), coastal and inland populations (*t*_(30,28)_ = −3.83, adj = 0.031), but not between the intermediate and inland populations (*t*_(28,27)_ = 0.69, *P* adj > 0.05).

### Effect of salinity on development rates

**Fig. 2:**
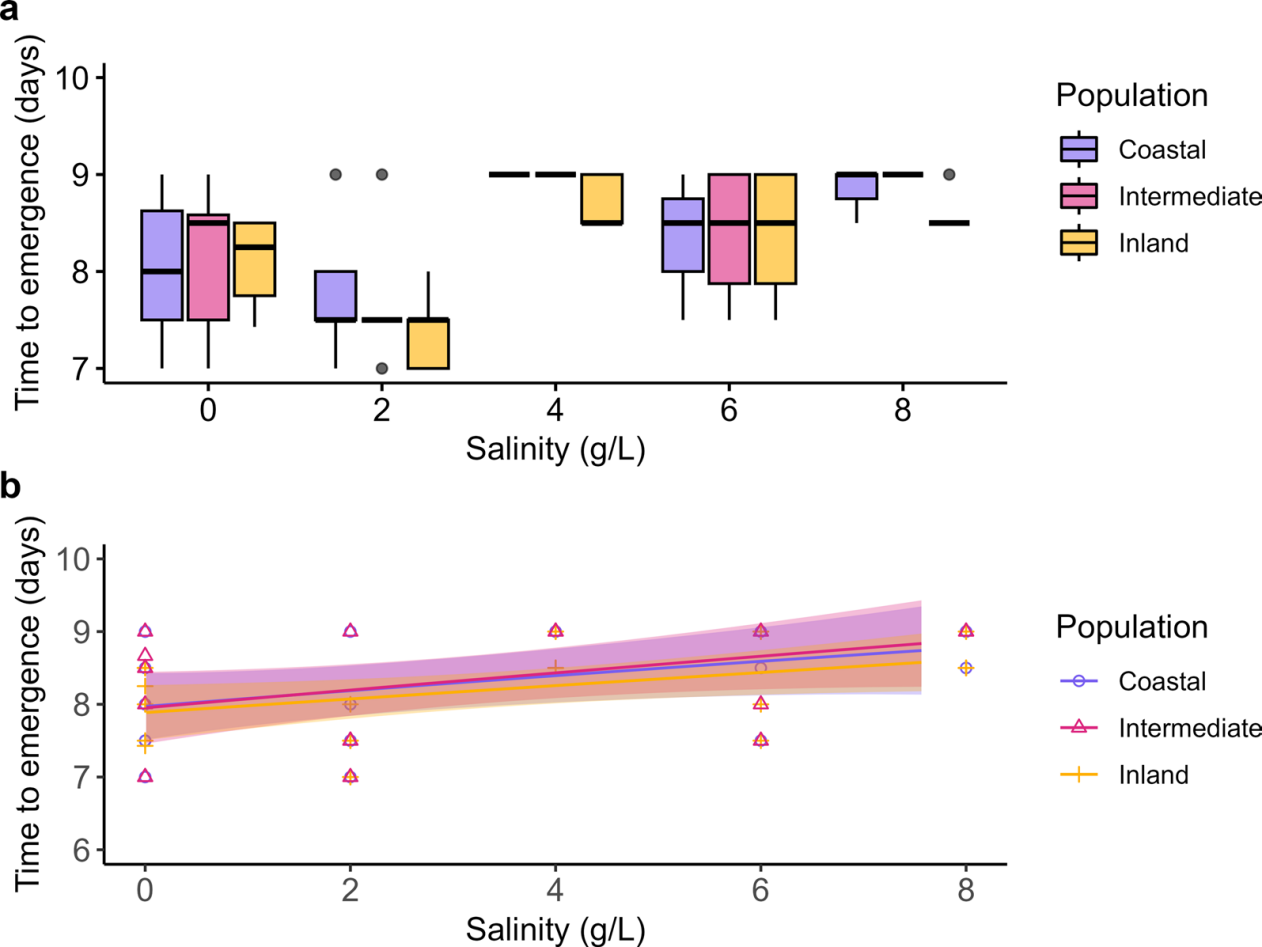
Normalized median time to emergence in days per population across increasing salinization levels as **a** boxplot with outliers as dots and **b** dose–response curve with standard error

A minor increase in the time to pupation (Additional file 1: Fig. S1) and time to emergence (Fig. 2) was detected with increasing salinity. Development to emergence was equally slowed for all populations. On average, larvae exposed to 8 g/l NaCl took 1 day longer to emerge than those exposed to 0 g/l NaCl (*t*(4,71) = −2.849, *p* < 0.041, partial η^2^ = 0.412; Fig. 2; Additional file 1: Table S3).

### Effect of salinity on sex ratio

**Fig. 3:**
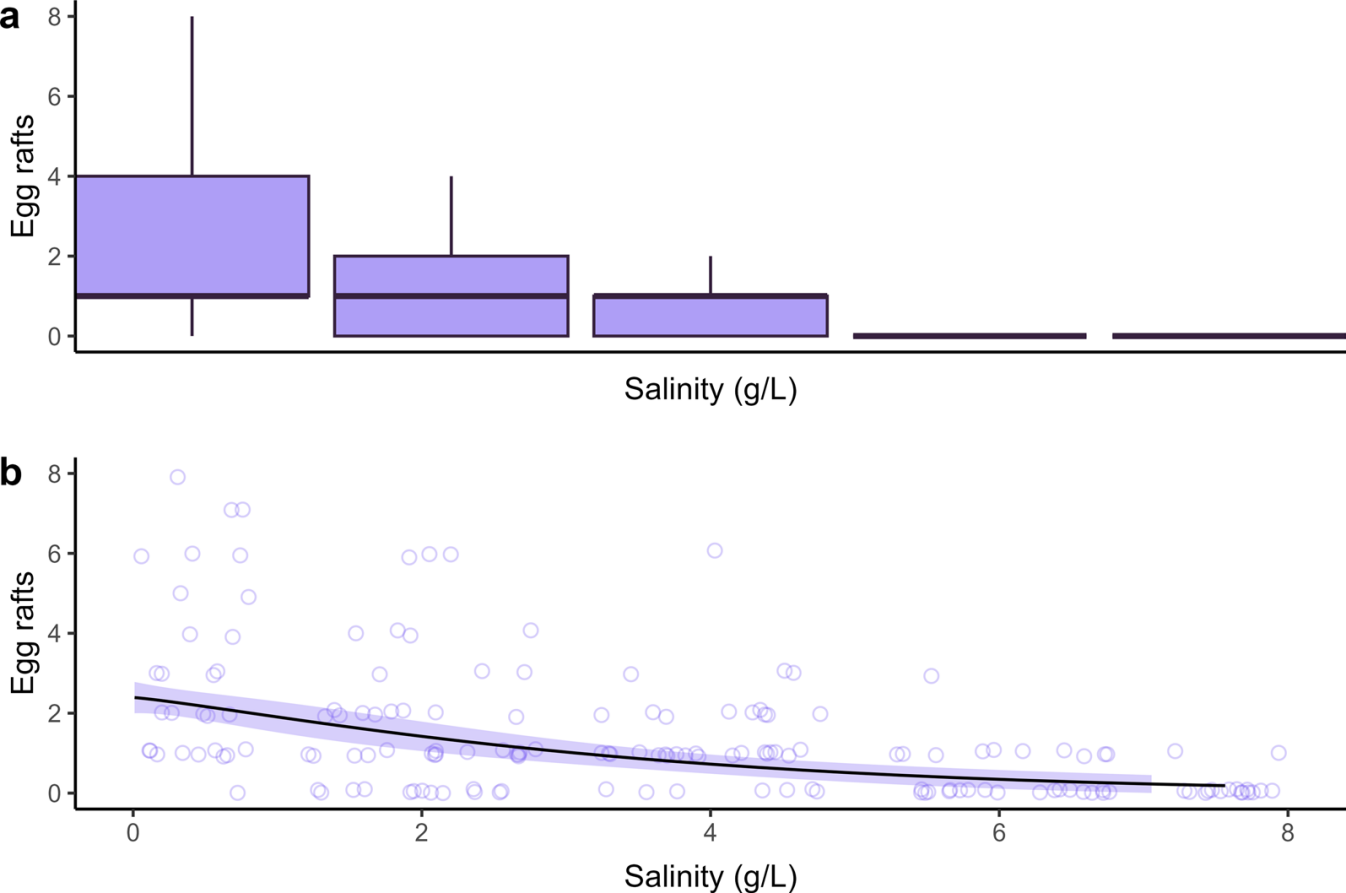
Daily ovipositioning behavior across increasing salinization levels, showing the number of egg rafts for each salinization level as **a** boxplot and **b** dose–response curve with standard error

A minor difference in sex ratio was detected with increasing salinity or among any of the populations (F(2,62) = 3.266, *p* = 0.045, partial η^2^ = 0.102; Fig. 3; Additional file 1: Table S4), between the coastal and inland populations (*P* adj = 0.013).

### Effect of salinity on ovipositioning behavior

Oviposition decreased with increasing salt concentration (*F*_(4297)_ = 25.863, *P* < 0.001, partial η^2^ = 0.273; Fig. 3; Table 2; Additional file 1: Table S5). The average oviposition rate decreased by 67% to 1.5 rafts or approximately 300 eggs [53] at 2 g/l and subsequently by 11% to one raft or approximately 200 eggs at 4 g/l. Oviposition rates at 6 g/l were almost negligible, at 9% (Additional file 1).

**Table 2:** Summary statistics on the ovipositioning rates for each salinity comparison

Contrast	Estimate	SE	*t* ratio	Adj. *P*-value
0 g/l–2 g/l	1.52	0.665	2.285	0.1997
0 g/l–4 g/l	1.92	0.667	2.879	0.0700
0 g/l–6 g/l	4.69	0.665	7.058	< 0.0001*
0 g/l–8 g/l	5.79	0.665	8.711	< 0.0001*
2 g/l–4 g/l	0.4	0.663	0.604	0.9724
2 g/l–6 g/l	3.17	0.661	4.802	0.0016*
2 g/l–8 g/l	4.27	0.661	6.464	0.0001*
4 g/l–6 g/l	2.77	0.663	4.183	0.0055*
4 g/l–8 g/l	3.87	0.663	5.841	0.0002*
6 g/l–8 g/l	1.1	0.661	1.663	0.4824

*Statistically significant

## Discussion

Contrary to our expectations, our results suggest that the investigated populations of *Cx pipiens* are highly tolerant to salinization, irrespective of their proximity to the current coastline. At the highest salinity (Fig. 1), representative of almost half the concentration of seawater, more than half of the larvae survived for all tested populations, instead of the expected 0% [15–17]. Differences in development rates among populations were observed, but the influence of salinity was evident only at 4 g/l or higher, resulting in a minor delay (Fig. 2). The sex ratios remained unaffected across the tested range, indicating no expected effect on potential population growth (Fig. 3). Our data additionally suggest that, although concentrations up to the previously described LD_50_ (4 g/l) were favored during egg laying, *Cx. pipiens* readily lays eggs under conditions of up to 6 g/l Cl^−^ and, incidentally, under 8 g/l Cl^−^. This finding is in line with observational data, as *Cx. pipiens* has recently been repeatedly observed to inhabit Dutch salt marches (pers. comm. J.G. van der Beek), which suggests a more congruent link between ovipositioning behavior and larval survival than has been described for other species [42, 57, 58].

Our observations are striking in contrast to the previously described LD_100_ of 6–7 g/l Cl^−^ in the USA and France [15–17]. There are several methodological differences between the current study and previous literature: (i) the use of second-instar larvae, which might increase the potential for physiological changes in response to saline conditions [34] relative to the use of older larvae; (ii) the use of eutrophic conditions, which, by increasing the energy budget of the larvae, might allow for higher metabolic rates, increasing the ability to expel the ionic waste [35]; and, finally, (iii) gradual acclimation of the locally sourced microbial community, which might have allowed for a higher microbial abundance and thus food availability during the experiment. The latter might have allowed for increased uptake of organic compounds, which may reduce the effects of the water’s osmolality [36]. While the relevance of each of these differences in setup cannot be distinguished with the current setup, the difference in total survival between our study and the earlier findings is far greater than might be explained by changes in methodology.

As our experimental setting is more representative of field conditions, the currently described responses might be more ecologically relevant than those described in previous studies under controlled conditions in the laboratory, as these generally use alternate food sources (e.g., fish feed), tap water without a natural microbial community [59], or laboratory-reared communities of a laboratory colony with a single subspecies. Given the ecological relevance of the setup applied, the observed pattern might be representative of populations in the Netherlands and possibly even for many other low-lying deltas. Based on these results, we speculate that similar patterns may exist for other mosquito species that inhabit lowland delta areas, such as *Culiseta morsitans*, *Culex modestus*, and perhaps even *Aedes aegypti*, which would imply that the current LD_50_ and LD_100_ should be reassessed. Taken together, the difference in the responses of our study and laboratory studies suggests that, while a wide range of mosquito species are typically associated with freshwater systems [60], they may exhibit substantial plasticity and/or (local) adaptation to increasing salinization.

## Conclusions

The current results suggest that coastal house mosquito populations will persist and will not show salinity-induced inland dispersal or local reductions in survival. The ecological implications are that they may instead locally increase in population size, despite the presence of predators. Many freshwater predator groups, including dragonflies and damselflies [61] and mayflies and true bugs [62], have longer generation times and may be vulnerable to salinization within the range tested. However, this assumption remains to be tested. Species diversity in transitory systems tends to decrease between freshwater and saline water [5, 18], while total insect abundance may remain unchanged [19]. Consequently, species that are able to persist in such systems may experience alleviation of predation pressure, causing population sizes to increase over time and increasing nuisance and disease risk. However, additional information is needed, as many studies on the tolerance of predator species are prone to methodological limitations similar to those of prior work on mosquitoes themselves. Nevertheless, house mosquito nuisance in coastal areas is likely to persist during the foreseeable future, and our results suggest that it is not unlikely that other mosquito species in coastal areas are similarly able to adapt to increasing salt levels even though their predators cannot.

### Supplementary Information

**Additional file 1.** Electronic appendix.

**Additional file 2.** Original data.

**Additional file 3.** Script for statistical analyses.

## Data Availability

Data supporting the conclusions of this article are included within the article and its appendices. The original datasets used and analyzed during the present study are freely and openly available within the appendices. All data generated or analysed during this study are included in this published article and its supplementary information files 2, 3.
