# Supplementary material for: Taking it with a grain of salt: tolerance to increasing salinization in Culex pipiens (Diptera: Culicidae) across a low-lying delta
Source: Parasit Vectors. 2024 Jun 10;17:251. doi: 10.1186/s13071-024-06268-8 (PMC11165877; doi:10.1186/s13071-024-06268-8)
Supplement: Supplementary file 1 — Additional file 1. Electronic appendix. [file 13071_2024_6268_MOESM1_ESM.docx]

**Electronic appendix**

**Time to pupation**

Lambda = 2

Formula: ((Day^lambda - 1)/lambda) ~ Treatment + (1 | Cosm)

Data: DR_MTP

Analysis of Variance Table

npar Sum Sq Mean Sq F value

Treatment 4 2006.8 501.7 7.2123

REML criterion at convergence: 1608

Scaled residuals:

Min 1Q Median 3Q Max

-2.4045 -0.4825 0.2376 0.6180 2.1521

Random effects:

Groups Name Variance Std.Dev.

Cosm (Intercept) 0.00 0.00

Residual 72.94 8.54

Number of obs: 228, groups: Cosm, 45

Fixed effects:

Estimate Std. Error t value

(Intercept) 28.0353 0.9263 30.265

Treatment2 -6.4149 1.4862 -4.316

Treatment4 7.4111 1.8609 3.983

Treatment6 1.4353 1.7330 0.828

Treatment8 6.6869 1.8866 3.544

Correlation of Fixed Effects:

(Intr) Trtmn2 Trtmn4 Trtmn6

Treatment2 - 0.623

Treatment4 - 0.498 0.310

Treatment6 -0.535 0.333 0.266

Treatment8 -0.491 0.306 0.244 0.262

optimizer (nloptwrap) convergence code: 0 (OK)

(Intercept) Treatment2 Treatment4 Treatment6 Treatment8

1.479537e-80 2.393689e-05 9.253775e-05 4.084389e-01 4.802100e-04

contrast estimate SE df t.ratio p.value

Treatment0 - Treatment2 6.415 1.49 48.5 4.299 0.0008

Treatment0 - Treatment4 -7.411 1.86 114.8 -3.977 0.0011

Treatment0 - Treatment6 -1.435 1.74 83.3 -0.825 0.9221

Treatment0 - Treatment8 -6.687 1.89 118.0 -3.540 0.0051

Treatment2 - Treatment4 -13.826 1.99 200.8 -6.936 <.0001

Treatment2 - Treatment6 -7.850 1.88 101.5 -4.183 0.0006

Treatment2 - Treatment8 -13.102 2.02 134.3 -6.497 <.0001

Treatment4 - Treatment6 5.976 2.18 159.6 2.737 0.0530

Treatment4 - Treatment8 0.724 2.30 183.5 0.314 0.9979

Treatment6 - Treatment8 -5.252 2.20 194.7 -2.382 0.1245

Note: contrasts are still on the (scale

Degrees-of-freedom method: kenward-roger

P value adjustment: tukey method for comparing a family of 5 estimates

**Time to emergence**

lambda = 2

Formula: ((Day^lambda - 1)/lambda) ~ Treatment + (1 | Cosm)

REML criterion at convergence: 1256

Analysis of Variance Table

npar Sum Sq Mean Sq F value

Treatment 4 2006.8 501.7 7.2123

Scaled residuals:

Min 1Q Median 3Q Max

-2.24368 -0.80490 0.09433 0.95199 1.45162

Random effects:

Groups Name Variance Std.Dev.

Cosm (Intercept) 0.00 0.00

Residual 69.56 8.34

Number of obs: 180, groups: Cosm, 44

Fixed effects:

Estimate Std. Error t value

(Intercept) 30.713 1.011 30.366

Treatment2 -2.820 1.505 -1.874

Treatment4 7.587 2.379 3.189

Treatment6 1.347 1.951 0.690

Treatment8 6.631 2.317 2.861

Correlation of Fixed Effects:

(Intr) Trtmn2 Trtmn4 Trtmn6

Treatment2 -0.672

Treatment4 -0.425 0.286

Treatment6 -0.518 0.348 0.220

Treatment8 -0.436 0.293 0.186 0.226

optimizer (nloptwrap) convergence code: 0 (OK)

(Intercept) Treatment2 Treatment4 Treatment6 Treatment8

3.139731e-71 6.262380e-02 1.695946e-03 4.908814e-01 4.742289e-03

contrast estimate SE df t.ratio p.value

Treatment0 - Treatment2 2.820 1.51 37.4 1.864 0.3541

Treatment0 - Treatment4 -7.587 2.39 129.2 -3.172 0.0160

Treatment0 - Treatment6 -1.347 1.97 60.0 -0.685 0.9591

Treatment0 - Treatment8 -6.631 2.33 116.2 -2.849 0.0407

Treatment2 - Treatment4 -10.407 2.44 160.6 -4.273 0.0003

Treatment2 - Treatment6 -4.167 2.02 66.9 -2.062 0.2486

Treatment2 - Treatment8 -9.451 2.37 122.5 -3.983 0.0011

Treatment4 - Treatment6 6.240 2.74 133.5 2.277 0.1590

Treatment4 - Treatment8 0.956 3.01 160.6 0.318 0.9978

Treatment6 - Treatment8 -5.284 2.69 174.0 -1.968 0.2862

Note: contrasts are still on the (scale

Degrees-of-freedom method: kenward-roger

P value adjustment: tukey method for comparing a family of 5 estimates

**Sex-ratio**

Lambda = 0.3838384

Formula: ((SRlog_corrected_rel^lambda - 1)/lambda) ~ City + (1 | Cosm)

REML criterion at convergence: 151

Analysis of Variance Table

npar Sum Sq Mean Sq F value

City 2 0.23147 0.11573 3.0896

Scaled residuals:

Min 1Q Median 3Q Max

-1.43023 -0.85746 0.08101 0.65570 2.71416

Random effects:

Groups Name Variance Std.Dev.

Cosm (Intercept) 0.0000 0.0000

Residual 0.6493 0.8058

Number of obs: 62, groups: Cosm, 45

Fixed effects:

Estimate Std. Error t value

(Intercept) -1.9143 0.1758 -10.887

Intermediate 0.1141 0.2487 0.459

Inland 0.4615 0.2518 1.833

Correlation of Fixed Effects:

(Intr) CtyUtr

Intermediate -0.707

Inland -0.698 0.494

optimizer (nloptwrap) convergence code: 0 (OK)

(Intercept) Intermediate Inland

1.520655e-15 6.481710e-01 7.199709e-02

**Total proportion of survival**

Lambda = 0.5858586

Formula: ((ASR_corrected_rel^lambda - 1)/lambda) ~ City + Treatment + (1 | Cosm)

REML criterion at convergence: 36.4

npar Sum Sq Mean Sq F value

City 2 0.63438 0.31719 8.2936

Treatment 4 0.85740 0.21435 5.6047

Scaled residuals:

Min 1Q Median 3Q Max

-2.06181 -0.55392 -0.00123 0.56390 2.81515

Random effects:

Groups Name Variance Std.Dev.

Cosm (Intercept) 0.01721 0.1312

Residual 0.05761 0.2400

Number of obs: 85, groups: Cosm, 45

Fixed effects:

Estimate Std. Error t value

(Intercept) -0.66000 0.06881 -9.591

Intermediate 0.20807 0.07248 2.871

Inland 0.23742 0.06967 3.408

Treatment2 0.14136 0.09428 1.499

Treatment4 -0.05414 0.09205 -0.588

Treatment6 -0.33562 0.09216 -3.642

Treatment8 -0.22498 0.09021 -2.494

Correlation of Fixed Effects:

(Intr) Intermediate CtyNjm Trtmn2 Trtmn4 Trtmn6

Intermediate -0.510

Inland -0.485 0.481

Treatment2 -0.467 -0.007 -0.051

Treatment4 -0.497 -0.003 0.005 0.493

Treatment6 -0.499 0.027 -0.022 0.364 0.371

Treatment8 -0.501 -0.002 -0.016 0.371 0.379 0.517

(Intercept) Intermediate Inland Treatment2 Treatment4 Treatment6 Treatment8

9.914777e-15 5.302853e-03 1.050250e-03 1.379366e-01 5.582053e-01 4.919582e-04 1.480756e-02

contrast estimate SE df t.ratio p.value

Coastal - Intermediate -0.2081 0.0744 78.0 -2.798 0.0176

Coastal - Inland -0.2374 0.0713 69.7 -3.332 0.0039

Intermediate - Inland -0.0294 0.0742 77.5 -0.395 0.9175

Results are averaged over the levels of: Treatment

Note: contrasts are still on the ( scale

Degrees-of-freedom method: kenward-roger

P value adjustment: tukey method for comparing a family of 3 estimates

contrast estimate SE df t.ratio p.value

Treatment0 - Treatment2 -0.1414 0.0945 63.9 -1.496 0.5691

Treatment0 - Treatment4 0.0541 0.0922 62.4 0.587 0.9765

Treatment0 - Treatment6 0.3356 0.0923 62.3 3.637 0.0049

Treatment0 - Treatment8 0.2250 0.0902 60.8 2.493 0.1056

Treatment2 - Treatment4 0.1955 0.0942 41.6 2.076 0.2494

Treatment2 - Treatment6 0.4770 0.1054 75.3 4.526 0.0002

Treatment2 - Treatment8 0.3663 0.1037 74.9 3.533 0.0062

Treatment4 - Treatment6 0.2815 0.1035 74.9 2.719 0.0605

Treatment4 - Treatment8 0.1708 0.1017 74.5 1.680 0.4521

Treatment6 - Treatment8  -0.1106 0.0897 38.5 -1.233 0.7323

Results are averaged over the levels of: City

Note: contrasts are still on the ( scale

Degrees-of-freedom method: kenward-roger

P value adjustment: tukey method for comparing a family of 5 estimates

*slope*

group1 group2 n1 n2 statistic df p p.adj

Leiden Nijmegen 30 28 -3.8344723 55.9619 0.000321 0.000963

Leiden Utrecht 30 27 -2.5086395 49.80637 0.015 0.031

Nijm. Utrecht 28 27 0.6902263 47.41384 0.493 0.493

**Ovipositioning behavior**

Lambda = -0.1818182

Formula: ((Egg_rafts^lambda - 1)/lambda) ~ Treatment + (1 | Location) + (1 | Day) + (1 | Cosm)

Random effects: REML criterion at convergence: 1504.2

Analysis of Variance Table

npar Sum Sq Mean Sq F value

Treatment 4 887.19 221.8 25.863

Scaled residuals:

Min 1Q Median 3Q Max

-2.23494 -0.70171 0.00891 0.77327 2.20855

Random effects:

Groups Name Variance Std.Dev.

Cosm (Intercept) 0.3772 0.6142

Day (Intercept) 0.8563 0.9254

Location (Intercept) 0.6625 0.8139

Residual 8.5758 2.9284

Number of obs: 297, groups: Cosm, 25; Day, 12; Location, 5

Fixed effects:

Estimate Std. Error t value

(Intercept) -1.1741 0.6538 -1.796

Treatment2 -1.5191 0.6648 -2.285

Treatment4 -1.9195 0.6666 -2.880

Treatment6 -4.6923 0.6648 -7.059

Treatment8 -5.7911 0.6648 -8.711

Correlation of Fixed Effects:

(Intr) Trtmn2 Trtmn4 Trtmn6

Treatment2 -0.514

Treatment4 -0.513 0.504

Treatment6 -0.514 0.506 0.504

Treatment8 -0.514 0.506 0.504 0.506

(Intercept) Treatment2 Treatment4 Treatment6 Treatment8

7.357064e-02 2.303516e-02 4.280169e-03 1.260673e-11 2.397374e-16

contrast estimate SE df t.ratio p.value

Treatment0 - Treatment2 1.52 0.665 16.1 2.285 0.1997

Treatment0 - Treatment4 1.92 0.667 16.3 2.879 0.0700

Treatment0 - Treatment6 4.69 0.665 16.1 7.058 <.0001

Treatment0 - Treatment8 5.79 0.665 16.1 8.711 <.0001

Treatment2 - Treatment4 0.40 0.663 16.0 0.604 0.9724

Treatment2 - Treatment6 3.17 0.661 15.8 4.802 0.0016

Treatment2 - Treatment8 4.27 0.661 15.8 6.464 0.0001

Treatment4 - Treatment6 2.77 0.663 16.0 4.183 0.0055

Treatment4 - Treatment8 3.87 0.663 16.0 5.841 0.0002

Treatment6 - Treatment8 1.10 0.661 15.8 1.663 0.4824

Note: contrasts are still on the ( scale

Degrees-of-freedom method: kenward-roger

P value adjustment: tukey method for comparing a family of 5 estimates

Table S1 Differences in survival rate over the salinity gradient

| Contrast |  | Estimate | SE | Df | T.ratio |
| --- | --- | --- | --- | --- | --- |
| Coastal - intermediate | -0.2081 | 0.0744 | 78.0 | -2.798 | 0.0176 |
| Coastal - inland | -0.2374 | 0.0713 | 69.7 | -3.332 | 0.0039 |
| Intermediate - inland | -0.0294 | 0.0742 | 77.5 | -0.395 | 0.9175 |

Table S2 Summary statistics on the survival ratios for each salinity comparison per population

| Population | Coastal |  |  |  |  |  |  |  |
| --- | --- | --- | --- | --- | --- | --- | --- | --- |
|  | Contrast |  |  | Estimate | SE | Df | T.ratio | P.value |
|  | 0 g/L | - | 2 g/L | -0.131 | 0.095 | 62.9 | -1.378 | 0.6437 |
|  | 0 g/L | - | 4 g/L | 0.0714 | 0.0951 | 63.2 | 0.751 | 0.9434 |
|  | 0 g/L | - | 6 g/L | 0.3519 | 0.0927 | 61.5 | 3.796 | 0.003 |
|  | 0 g/L | - | 8 g/L | 0.2125 | 0.0927 | 61.7 | 2.293 | 0.1611 |
|  | 2 g/L | - | 4 g/L | 0.2023 | 0.0998 | 41.2 | 2.028 | 0.2711 |
|  | 2 g/L | - | 6 g/L | 0.4828 | 0.1061 | 73.3 | 4.551 | 0.0002 |
|  | 2 g/L | - | 8 g/L | 0.3434 | 0.1061 | 73.3 | 3.237 | 0.0152 |
|  | 4 g/L | - | 6 g/L | 0.2805 | 0.1064 | 73.3 | 2.635 | 0.0744 |
|  | 4 g/L | - | 8 g/L | 0.1411 | 0.1064 | 73.5 | 1.327 | 0.6757 |
|  | 6 g/L | - | 8 g/L | -0.1394 | 0.0946 | 37.4 | -1.473 | 0.5857 |
|  |  |  |  |  |  |  |  |  |
| Population | Intermediate |  |  |  |  |  |  |  |
|  | Contrast |  |  | Estimate | SE | Df | T.ratio | P.value |
|  | 0 g/L | - | 2 g/L | -0.131 | 0.095 | 62.9 | -1.378 | 0.6437 |
|  | 0 g/L | - | 4 g/L | 0.0714 | 0.0951 | 63.2 | 0.751 | 0.9434 |
|  | 0 g/L | - | 6 g/L | 0.3519 | 0.0927 | 61.5 | 3.796 | 0.003 |
|  | 0 g/L | - | 8 g/L | 0.2125 | 0.0927 | 61.7 | 2.293 | 0.1611 |
|  | 2 g/L | - | 4 g/L | 0.2023 | 0.0998 | 41.2 | 2.028 | 0.2711 |
|  | 2 g/L | - | 6 g/L | 0.4828 | 0.1061 | 73.3 | 4.551 | 0.0002 |
|  | 2 g/L | - | 8 g/L | 0.3434 | 0.1061 | 73.3 | 3.237 | 0.0152 |
|  | 4 g/L | - | 6 g/L | 0.2805 | 0.1064 | 73.3 | 2.635 | 0.0744 |
|  | 4 g/L | - | 8 g/L | 0.1411 | 0.1064 | 73.5 | 1.327 | 0.6757 |
|  | 6 g/L | - | 8 g/L | -0.1394 | 0.0946 | 37.4 | -1.473 | 0.5857 |
|  |  |  |  |  |  |  |  |  |
| Population | Inland |  | |  |  |  |  |  |
|  | Contrast |  |  | Estimate | SE | Df | T.ratio | P.value |
|  | 0 g/L | - | 2 g/L | -0.131 | 0.095 | 62.9 | -1.378 | 0.6437 |
|  | 0 g/L | - | 4 g/L | 0.0714 | 0.0951 | 63.2 | 0.751 | 0.9434 |
|  | 0 g/L | - | 6 g/L | 0.3519 | 0.0927 | 61.5 | 3.796 | 0.003 |
|  | 0 g/L | - | 8 g/L | 0.2125 | 0.0927 | 61.7 | 2.293 | 0.1611 |
|  | 2 g/L | - | 4 g/L | 0.2023 | 0.0998 | 41.2 | 2.028 | 0.2711 |
|  | 2 g/L | - | 6 g/L | 0.4828 | 0.1061 | 73.3 | 4.551 | 0.0002 |
|  | 2 g/L | - | 8 g/L | 0.3434 | 0.1061 | 73.3 | 3.237 | 0.0152 |
|  | 4 g/L | - | 6 g/L | 0.2805 | 0.1064 | 73.3 | 2.635 | 0.0744 |
|  | 4 g/L | - | 8 g/L | 0.1411 | 0.1064 | 73.5 | 1.327 | 0.6757 |
|  | 6 g/L | - | 8 g/L | -0.1394 | 0.0946 | 37.4 | -1.473 | 0.5857 |


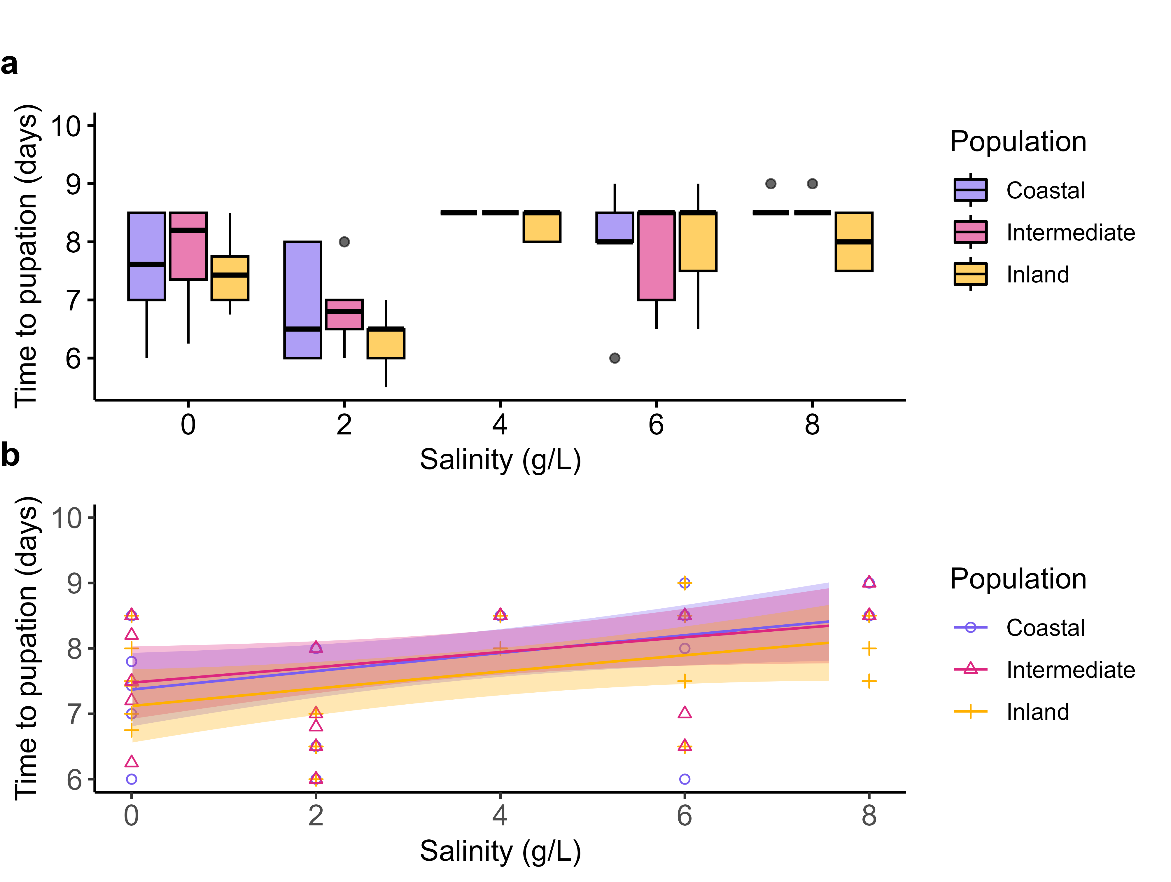


Figure S1 Normalized median time to pupation in days per population across increasing salinization levels as a. boxplot with outliers as dots and b. dose-response curve with standard error.


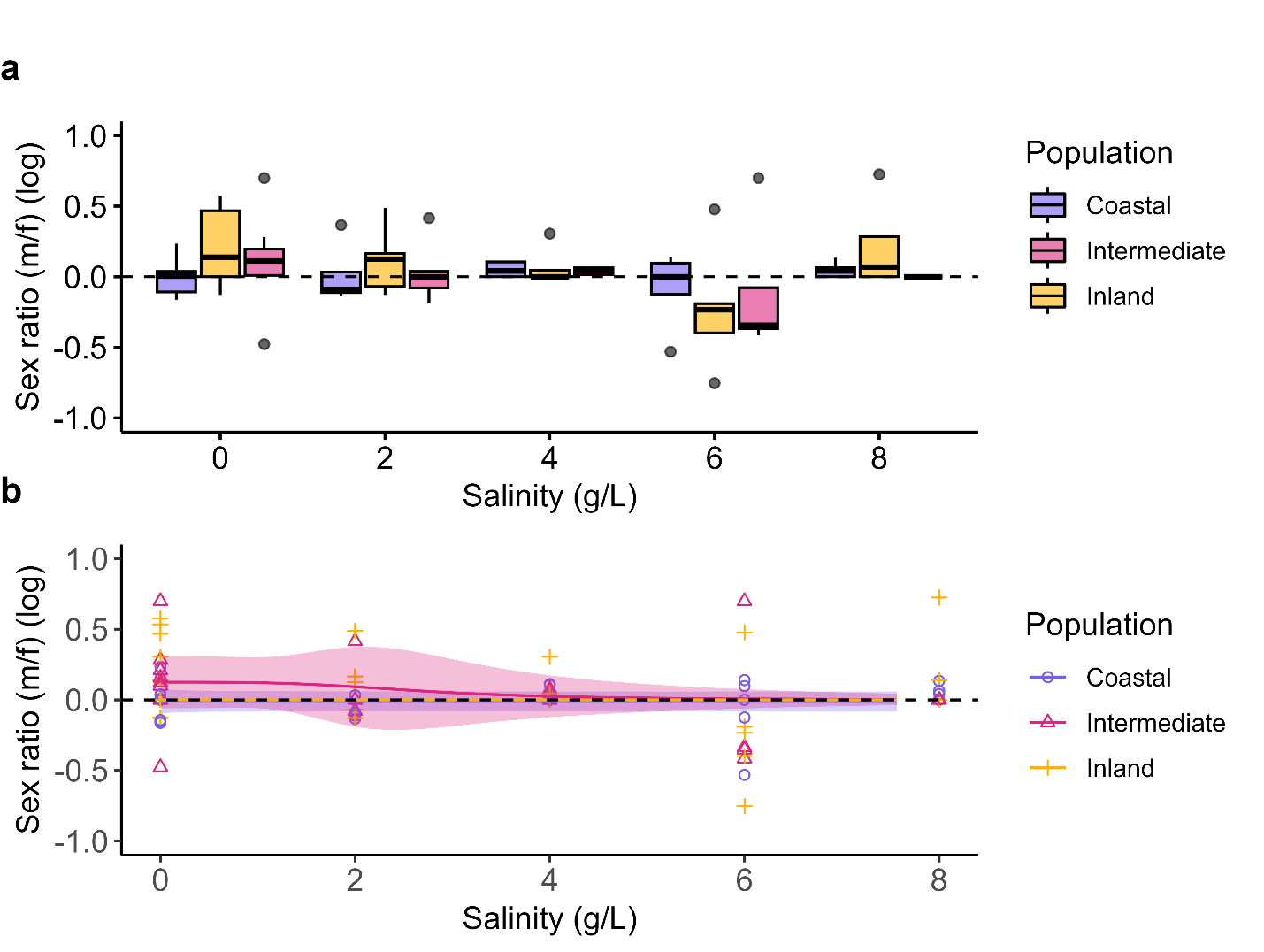


Figure S2 Normalized male to female sex ratio (transformed as natural logarithm) at the end of the experiment per population across increasing salinization levels as a. boxplot with outliers as dots and b. dose-response curve with standard error

Table S3 Model summary statistics on the time to emergence for each salinity comparison and population.

|  | Estimate | Std. Error | t value | p value |
| --- | --- | --- | --- | --- |
| (Intercept) | 30.713 | 1.011 | 30.366 | 3.14e-71 |
| Treatment2 | -2.82 | 1.505 | -1.874 | 6.26e-02 |
| Treatment4 | 7.587 | 2.379 | 3.189 | 1.70e-03 |
| Treatment6 | 1.347 | 1.951 | 0.69 | 4.91e-01 |
| Treatment8 | 6.631 | 2.317 | 2.861 | 4.74e-03 |

Table S4 Model summary statistics on the male:female sex ratio for each population

|  | Estimate | Std. Error | t value | p value |
| --- | --- | --- | --- | --- |
| (Intercept) | -1.9143 | 0.1758 | -10.887 | 1.52E-15 |
| Inland population | 0.1141 | 0.2487 | 0.459 | 6.48E-01 |
| Intermediate population | 0.4615 | 0.2518 | 1.833 | 7.20E-02 |

Table S5 Model summary statistics on the ovipositioning behavior for each population

|  | Estimate | Std. Error | t value | p value |
| --- | --- | --- | --- | --- |
| (Intercept) | -1.1741 | 0.6538 | -1.796 | 7.36E-02 |
| Treatment2 | -1.5191 | 0.6648 | -2.285 | 2.30E-02 |
| Treatment4 | -1.9195 | 0.6666 | -2.88 | 4.28E-03 |
| Treatment6 | -4.6923 | 0.6648 | -7.059 | 1.26E-11 |
| Treatment8 | -5.7911 | 0.6648 | -8.711 | 2.40E-16 |
